# Creativity Beyond the Algorithm: Aortic Aneurysm Repair in an LVAD Patient

**DOI:** 10.1016/j.jaccas.2026.109325

**Published:** 2026-09-02

**Authors:** Maciej Kolowca

**Affiliations:** Cardiac Surgery Department, University of Rzeszow, Rzeszow, Poland

**Keywords:** aorta, cardiac assist devices, vascular disease

**Figure:**
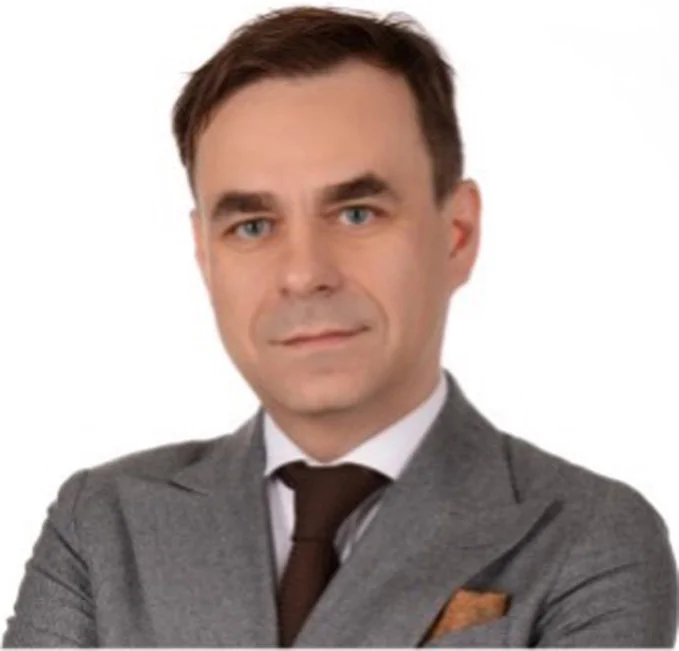

Although the number of new continuous-flow left ventricular assist device (LVAD) implantations has remained relatively stable over the past few years, the number of patients living with these devices continues to increase.^1^ This is mainly because of progressively longer survival, improved clinical outcomes, a lower incidence of complications, and a shift in treatment strategy toward a higher proportion of destination therapy. At the same time, patients in this population are becoming older, with a growing burden of comorbidities and increasing clinical complexity.

The case report presented by Kardes et al^2^ in the current issue of *JACC: Case Reports* provides an excellent illustration of the increasingly complex clinical scenarios we are now facing. Such cases require creative, nonstandard, and carefully individualized strategies to achieve the best possible clinical outcomes.

The authors describe an exceptionally complex case of successful treatment in a patient with an implanted LVAD. The patient had previously experienced an acute DeBakey type I aortic dissection and had undergone 3 interventions related to this condition: ascending aortic replacement in the acute phase, followed subsequently by frozen elephant trunk repair and thoracic endovascular aortic repair extension. Because of myocardial infarction and progressive left ventricular failure, a HeartWare LVAD was implanted. During further follow-up, the patient developed aneurysmal degeneration of a chronic aortic dissection, reaching a maximum diameter of 77 mm at the level of the abdominal aorta, just above the aortic bifurcation. The true lumen was extremely narrow, and the left renal artery originated from the false lumen.

Treatment of this life-threatening condition, caused by such a large dissecting aortic aneurysm, required an entirely nonstandard approach in this highly complex patient. To combine effective treatment with the least technically demanding endovascular solution, while at the same time preserving renal function and avoiding compromising the perfusion of the left kidney, the authors chose a truly innovative strategy: They performed autotransplantation of the left kidney into the pelvis, with reimplantation of the renal artery into the iliac artery. This allowed them to perform a rapid and effective endovascular aortic repair (EVAR) procedure, achieving complete exclusion of the large aneurysm while maintaining perfusion to both kidneys. After the procedure, the patient had preserved renal function and avoided the need for dialysis.

One of the most difficult and crucial aspects of safely guiding a patient with an implanted LVAD through a surgical procedure is appropriate management of anticoagulation. On the one hand, the patient must be protected from excessive perioperative bleeding; on the other hand, thrombus formation within the LVAD system and peripheral embolic complications must be avoided. This is particularly challenging in patients supported with the HeartWare system, which is known for higher burden of hemocompatibility-related adverse events,^3^ one of the factors that contributed to its withdrawal from the market. Nevertheless, many patients continue to live with well-functioning HeartWare devices, and this remains an important clinical challenge.

In the presented case, renal autotransplantation required 2 separate surgical accesses, precise vascular anastomoses, and ureteral reimplantation. The retroperitoneal space and pelvis are areas associated with a particularly high risk of bleeding, especially in a patient supported with an LVAD. The authors decided to perform the operation under ongoing antiplatelet therapy and low–molecular weight heparin, after discontinuation of oral anticoagulation and administration of prothrombin complex concentrate. This allowed them to achieve an international normalized ratio below 1.5. However, treatment with low–molecular weight heparin should be guided by anti-Xa activity rather than activated partial thromboplastin time. This perioperative period is particularly dangerous for patients supported with a HeartWare device. In this case, the chosen anticoagulation strategy proved effective. The authors did not report any bleeding complications at the surgical site, nor any thrombotic complications within the LVAD system or embolic events in the peripheral circulation.

The effort of Kardes et al^2^ to preserve the kidney and thereby avoid the risk of hemodialysis deserves particular emphasis. The need for chronic hemodialysis could substantially reduce quality of life in a patient supported with an LVAD and may also significantly limit survival.^4^ The autotransplantation procedure selected by the authors is, of course, associated with the risk of potential autograft failure. To perform this part of the operation safely, adequate organ protection is essential, preferably using several complementary strategies, including hypothermia and perfusion of the kidney with an organ-protective preservation solution.

The very challenging anatomical conditions within the dissected abdominal aorta forced the authors to perform EVAR in the simplest feasible manner. The use of branched stent grafts would have been extremely difficult to perform safely and effectively in this setting. Another potential option could have been a custom-made, physician-modified stent graft, with fenestration and scalloping performed on the back table during the procedure. However, in this case, the length of the proximal landing zone may not have been sufficient. The described patient developed distal stent graft–induced new entry (SINE) on 2 occasions: first after the frozen elephant trunk procedure and again after EVAR. The planned stent graft diameter and degree of oversizing should therefore be selected with great caution in such patients, particularly in the presence of chronic aortic dissection, LVAD support, renal dysfunction, and chronic anticoagulation. Personally, I would use oversizing of no more than 10%, based on measurement of the cross-sectional area of both the landing zone and the stent graft. The use of tapered stent grafts may also be helpful in selected patients. When choosing the degree of oversizing, we are always balancing the risk of SINE against the risk of endoleak or stent graft migration, however the first complication could be more malignant than the other two.

The most important lesson from this case is that such complex patients should be treated in highly specialized centers by a multidisciplinary team whose composition extends beyond even the well-established frameworks of the heart team or aortic team. Such an expanded team allows the development of the most appropriate treatment strategy for highly complex LVAD patients, such as the one described by Kardes et al.^2^ This team should define not only the general therapeutic strategy, but also the detailed elements that determine patient safety at every stage of care. In this case, anticoagulation management was one of the key components.

The presented case demonstrates, in an exemplary manner, how such clinically complex patients should be managed: through a combination of creativity, advanced technical expertise, and the collective experience of the entire therapeutic team.

## Funding Support and Author Disclosures

Dr Kolowca has been a proctor and medical consultant for Medtronic.
